# Supplementary material for: Calorimetric evidence for two phase transitions in Ba1−xKxFe2As2 with fermion pairing and quadrupling states
Source: Nat Commun. 2023 Oct 23;14:6734. doi: 10.1038/s41467-023-42459-0 (PMC10593811; doi:10.1038/s41467-023-42459-0)
Supplement: Supplementary file 1 — Supplementary Information [file 41467_2023_42459_MOESM1_ESM.pdf]

Supplementary Information: Calorimetric evidence for two phase transitions in $Ba_{1-x}K_xFe_2As_2$ with fermion pairing and quadrupling states

Ilya Shipulin[1,2,*], Nadia Stegani[3,4,*], Ilaria Maccari[5], Kunihiro Kihou[6], Chul-Ho Lee[6], Quanxin Hu[7], Yu Zheng[7], Fazhi Yang[7], Yongwei Li[7], Chi-Ming Yim[7,8], Ruben Hühne[1], Hans-Henning Klauss[2], Marina Putti[3,4], Federico Caglieris[3,4,9,+], Egor Babaev[5,†], and Vadim Grinenko[7,8,‡]

[1]Institute for Metallic Materials, Leibniz-IFW Dresden, D-01069, Dresden, Germany
[2]Institute for Solid State and Materials Physics, Technische Universität Dresden, D-01069 Dresden, Germany
[3]University of Genoa, Via Dodecaneso 33, 16146 Genoa, Italy
[4]Consiglio Nazionale delle Ricerche (CNR)-SPIN, Corso Perrone 24, 16152 Genova, Italy
[5]Department of Physics, KTH Royal Institute of Technology, SE-106 91 Stockholm, Sweden
[6]National Institute of Advanced Industrial Science and Technology (AIST), Tsukuba, Ibaraki 305-8568, Japan
[7]Tsung-Dao Lee Institute, Shanghai Jiao Tong University, Shanghai 201210, China
[8]School of Physics and Astronomy, Shanghai Jiao Tong University, Shanghai 200240, China
[9]Institute for Solid State Research, Leibniz-IFW Dresden, D-01069, Dresden, Germany
[+]federico.caglieris@spin.cnr.it
[†]babaev.egor@gmail.com
[‡]vadim.a.grinenko@gmail.com
[*]these authors contributed equally to this work

ABSTRACT

The supplementary information contains supplementary Figures S1 - S3.

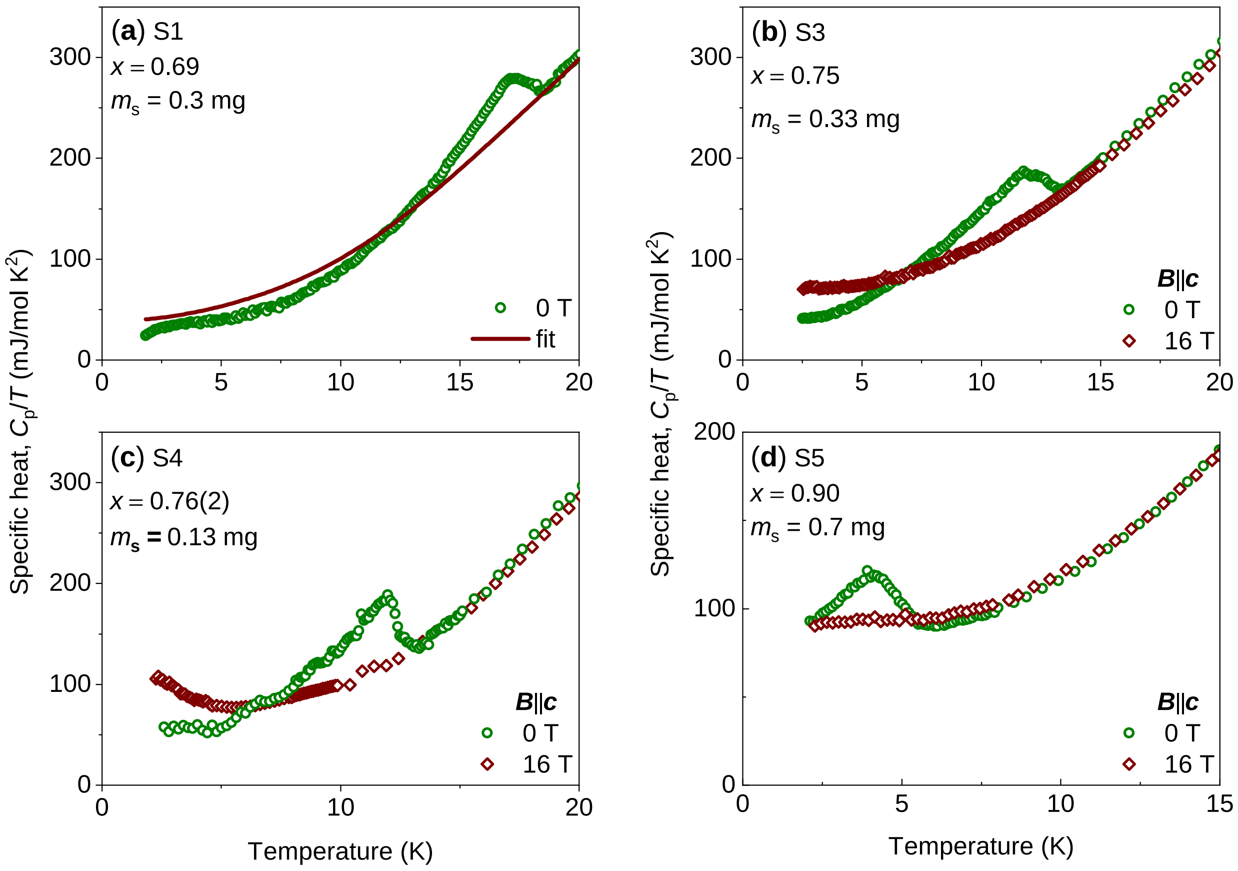

Figure S1. Raw specific heat data. Temperature dependence of the zero-field specific heat C_p/T for the $Ba_{1-x}K_xFe_2As_2$ samples $S1$, $S3$-$S5$ shown in the main text. The fitting curve in panel **(a)** is used to subtract the phonon background. The details of the fitting can be found in Ref.[1]. For samples $S3$-$S5$ shown in panels **(b-d)**, the data measured in 16 T field applied along the crystallographic c-axis were used to obtain the phonon background. The results of the subtraction are shown in Fig. 1.

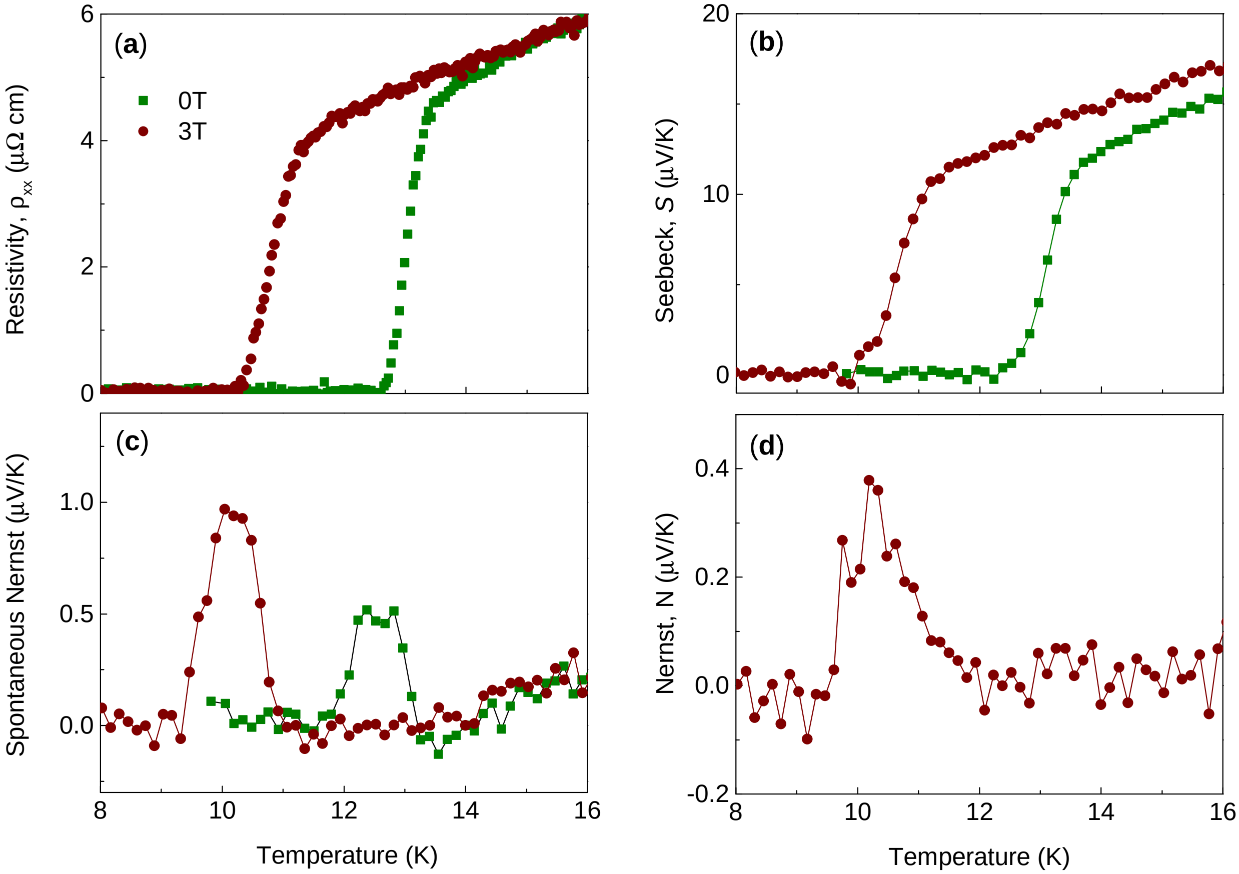

Figure S2. Transport data for the sample $S2$ **(a)** Temperature dependence of the longitudinal electrical resistivity close to superconducting transition measured in zero and 3 T field applied along the crystallographic c-axis. **(b)** Temperature dependence of the Seebeck effect. The resistivity and Seebeck effect have a very similar temperature dependence. **(c)** Temperature dependence of the spontaneous Nernst effect (even signal in a magnetic field). **(d)** Temperature dependence of the normal Nernst effect (odd signal in a magnetic field).

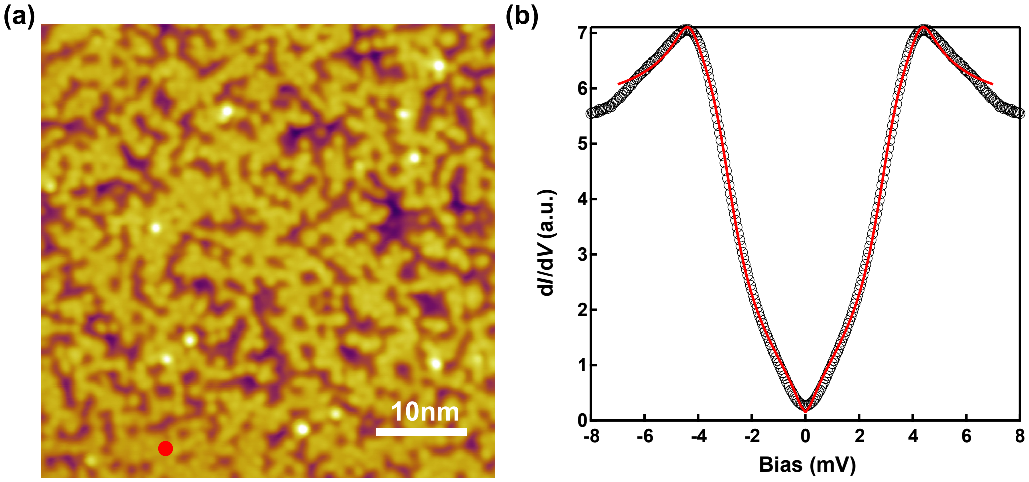

Figure S3. STM data for the sample S_{NP} (a) STM Topograph recorded from the disordered surface of a $\mathrm{Ba_{1\text{-}x}K_xFe_2As_2}$ sample with $x = 0.77$ (image size: $(50 \times 50)\mathrm{nm}^2$, $V_{\mathrm{b}} = 80\mathrm{mV}$, $I_{\mathrm{t}} = 100\mathrm{pA}$, $T = 0.3\mathrm{K}$). The disordered surface was likely caused by the mixture of Ba atoms and K atoms.**(b)** $\mathrm{d}I/\mathrm{d}V - V$ spectrum taken from a defect-free position (the red point) on the surface shown in (**a**) (Spectroscopic set-point: $V_s = 10\mathrm{mV}$, $I_s = 200\mathrm{pA}$, amplitude of bias modulation used $V_{\mathrm{mod}} = 0.25\mathrm{mV}$), showing a "V"-shaped superconducting gap. The black circle was experiment result. Red solid curve was fitting result using double-gap Dynes equation with a larger gap ($\Delta_2 = 3.1\mathrm{meV}$) and a smaller gap with node($\Delta_1 = 2.1\mathrm{meV}$). There is no "CDW" gap feature in the spectrum.

References

1. Grinenko, V. *et al.* Superconductivity with broken time-reversal symmetry inside a superconducting s-wave state. *Nat. Phys.* **16**, 789–794 (2020).
